# Seasonal Variation in Abundance and Diversity of Bacterial Methanotrophs in Five Temperate Lakes

**DOI:** 10.3389/fmicb.2017.00142

**Published:** 2017-02-03

**Authors:** Md Sainur Samad, Stefan Bertilsson

**Affiliations:** ^1^Department of Ecology and Genetics, Limnology and Science for Life Laboratory, Uppsala UniversityUppsala, Sweden; ^2^Department of Microbiology and Immunology, University of OtagoDunedin, New Zealand

**Keywords:** methanotrophs, methane, *pmoA*, qPCR, T-RFLP, lakes, ice cover

## Abstract

Lakes are significant sources of methane (CH_4_) to the atmosphere. Within these systems, methanotrophs consume CH_4_ and act as a potential biofilter mitigating the emission of this potent greenhouse gas. However, it is still not well understood how spatial and temporal variation in environmental parameters influence the abundance, diversity, and community structure of methanotrophs in lakes. To address this gap in knowledge, we collected water samples from three depths (surface, middle, and bottom) representing oxic to suboxic or anoxic zones of five different Swedish lakes in winter (ice-covered) and summer. Methanotroph abundance was determined by quantitative real time polymerase chain reaction and a comparison to environmental variables showed that temperature, season as well as depth, phosphate concentration, dissolved oxygen, and CH_4_ explained the observed variation in methanotroph abundance. Due to minimal differences in methane concentrations (0.19 and 0.29 μM for summer and winter, respectively), only a weak and even negative correlation was observed between CH_4_ and methanotrophs, which was possibly due to usage of CH_4_. Methanotrophs were present at concentrations ranging from 10^5^ to 10^6^ copies/l throughout the oxic (surface) and suboxic/anoxic (bottom) water mass of the lakes, but always contributed less than 1.3% to the total microbial community. Relative methanotroph abundance was significantly higher in winter than in summer and consistently increased with depth in the lakes. Phylogenetic analysis of *pmoA* genes in two clone libraries from two of the ice-covered lakes (Ekoln and Ramsen) separated the methanotrophs into five distinct clusters of *Methylobacter* sp. (Type I). Terminal restriction fragment length polymorphism analysis of the *pmoA* gene further revealed significant differences in methanotrophic communities between lakes as well as between winter and summer while there were no significant differences between water layers. The study provides new insights into diversity, abundance, community composition and spatial as well as temporal distribution of freshwater methanotrophs in low-methane dimictic lakes.

## Introduction

Methane (CH_4_) is a greenhouse gas which has a Global Warming Potential (GWP) 30 times higher than carbon dioxide (CO_2_) and it is also the second most important greenhouse gas after carbon dioxide (IPCC, 2013). CH_4_ production rates are known to depend on temperature, availability of organic matter, and absence of oxygen. The largest source of natural CH_4_ emission is from natural wetlands, which contributes 170 Tg/year (United States Environmental Protection Agency [USEPA], 2010). Freshwater lakes emit 5 Tg to the annual CH_4_ annually (Hanson and Hanson, 1996). The concentration of CH_4_ in lakes can vary dramatically. Juutinen et al. (2009) surveyed 207 Finnish lakes and reported surface and bottom water average methane concentrations of 1 and 20.6 μM, respectively, while the concentrations were <2.3 μM in 75% of the lakes. Bastviken et al. (2004) also reported surface water CH_4_ concentrations in summer of 11 North American lakes and 13 Swedish lakes, ranging from 0.27 to 2.32 μM and 0.08 to 1.89 μM, respectively. A resent study (Denfeld et al., 2016) also explored seven ice-covered Swedish lakes and measured CH_4_ concentrations in surface water samples which were in the range of 0.04–1.11 μM. Estimating CH_4_ emissions from lakes and reservoirs is difficult, since there are at least four emission pathways (ebullition, diffusive flux, storage flux, and flux through aquatic vegetation; Bastviken et al., 2004). The major part of CH_4_ that reaches the upper mixed zone of the water body is from diffusive flux and ebullition (Bastviken et al., 2004; Walter et al., 2007).

Methanotrophs is a functional guild of microorganisms that utilize CH_4_ as their sole carbon and energy source (Hanson and Hanson, 1996). They are found in a wide range of habitats (e.g., lakes, rivers, streams, oceans, ponds, sediments, mud, swamps, rice paddies, meadow soils, deciduous woods, sewage sludge, and several other environments; Hanson and Hanson, 1996; Trotsenko and Khmelenina, 2002) including humic (Taipale et al., 2009) and hypersaline lakes (Carini et al., 2005) and can grow at temperatures as low as 4°C (Bowman et al., 1997) or as high as 72°C (Bodrossy et al., 1999). Aerobic methanotrophs are historically classified into three major groups: Type I (*Methylobacter, Methylomonas, Methylosoma, Methylomicrobium, Methylothermus, Methylohalobius, Methylosarcina, Methylosphaera*), Type II (*Methylosinus, Methylocystis, Methylocella, Methylocapsa*), and Type X (*Methylococcus, Methylocaldum*), based on a combination of morphological characteristics, intracytoplasmic membrane structures, carbon assimilation pathways, ability to fix nitrogen, and some other physiological traits (Jiang et al., 2010; Park and Lee, 2013). In lakes, methanotrophs (Type I and Type II) are widely distributed in water and sediments (Costello and Lidstrom, 1999; Sundh et al., 2005; He et al., 2012; Osudar et al., 2016). It has been reported that the diversity of methanotrophs in lakes is greater than that found in peat or marine environments (Costello and Lidstrom, 1999). To date most of the studies of methanotrophs have focused on lake sediments as this functional guild is highly abundant in such systems (10^4^ to 10^7^ copies of *pmoA* per gram of dry sediment; Yang et al., 2016). Information about methanotroph abundance (via quantitative polymerase chain reaction, qPCR) in lake water columns is still scarce, but their presence has been demonstrated by several different methods: metagenome sequencing (Peura et al., 2015), proteomics (Ullrich et al., 2016), fluorescence *in situ* hybridization (Eller et al., 2005), and enrichment cultures (Lidstrom and Somers, 1984; He et al., 2012).

Oxidation of CH_4_ to methanol is the first enzymatic step in methanotroph metabolism. This process is catalyzed by the enzyme CH_4_ monooxygenase (MMO). There are two forms of MMO: a cytoplasmic version or soluble MMO (sMMO), and a membrane-bound version or particulate MMO (pMMO; Murrell et al., 2000). Almost all characterized methanotrophs feature either exclusively pMMO or both pMMO and sMMO. Members of the *Methylocella* genus are thus far the only methanotrophs where pMMO has not been detected (Dumont and Murrell, 2005). After transforming CH_4_ to methanol by the MMO, the methanol is oxidized to formaldehyde by a pyrroloquinoline quinone-dependent methanol dehydrogenase (Anthony and Dales, 1996; Jiang et al., 2010). At the level of formaldehyde, carbon is assimilated by either the ribulose monophosphate pathway (RuMP) or the serine pathway, depending on the organism (Hanson and Hanson, 1996; Jiang et al., 2010; Strong et al., 2015). Alternatively, formaldehyde can be oxidized completely to carbon dioxide, which creates reducing equivalents for cellular metabolism (Dumont and Murrell, 2005). Type I methanotrophs use the RuMP pathway, whereas Type II use the serine pathway. Type X methanotrophs use both pathways (mainly RuMP; Lieberman and Rosenzweig, 2004; Jiang et al., 2010; Park and Lee, 2013).

The *pmoA* gene encodes for a subunit of pMMO and it has been extensively used as a group-specific biomarker in molecular ecology studies of methanotrophs. This gene is present in all known methanotrophs with the exception of *Methylocella* and *Methyloferula* (Dedysh et al., 2000; Vorobev et al., 2011). There is a large database of *pmoA* gene sequences available from characterized methanotroph strains, which makes it easy to identify a methanotroph based on its *pmoA* gene sequence. There are a several PCR primers that can target *pmoA* with varying specificity (Dumont and Murrell, 2005). For example, the A198F/A682R primer set targets the *pmoA* gene and also the *amoA* gene in autotrophic ammonia oxidizers (Holmes et al., 1995). One often used *pmoA*-specific primer set is A189F/mb661R (Costello and Lidstrom, 1999). Because of its high specificity, it is possible to use these primers to quantify the abundance of methanotrophs using qPCR. This primer set is widely used in qPCR for quantitative study of methanotrophs (Shrestha et al., 2010; Xu et al., 2013).

Methanotrophs play a crucial role in controlling global warming as well as the global methane cycle (Conrad, 1996; Hanson and Hanson, 1996). Methane is mainly produced in anoxic sediments, wetlands, and waterlogged soils, and is subsequently utilized by methanotrophs during passage to the atmosphere. Hence, methanotrophs act as a biofilter for methane release to the atmosphere. These organisms may thus reduce the contribution of methane emissions to global warming (Hanson and Hanson, 1996; Park et al., 2002). However, it is still not well understood how environmental variables control or regulate the abundance, diversity, and community compositions of methanotrophs over spatial and temporal scales. The objectives of the present study were therefore: (1) to identify potential methanotroph hotspots in five Swedish lakes; (2) to describe how environmental variables are coupled to methanotroph abundance; and (3) to assess whether the methanotroph community vary over depth, between lakes and between seasons.

## Materials and Methods

### Study Sites and Sampling

Sampling was carried out in 2012 from five temperate lakes experiencing dimictic thermal stratification: Ekoln (59°46′>N, 17°37′>E), Erken (59°50′>N, 18°35′>E), Långsjön (60°01′>N, 17°34′>E), Siggeforasjön (59°58′>N, 17°09′>E), and Ramsen (59°49′>N, 17°54′>E). Samples were collected from three different depth layers of the water column: surface (1 m depth below the surface: the epilimnion zone), middle (the metalimnion zone or middle depth), and bottom [immediately above (∼1 m) sediment–water interfaces: the hypolimnion] of the lakes. The five lakes were all located in the eastern part of south-central Sweden (Supplementary Figure S1) and vary in size and trophic state (**Table 1**).

**Table 1:** Lake characteristics.

	Trophic state	Lake area (km^2^)	Maximum depth (m)	Reference
Ekoln	Eutrophic	20	40	Eiler and Bertilsson, 2004
Erken	Mesotrophic	24	21	Elliott et al., 2007
Långsjön	Oligotrophic	2.5	12.5	Quevedo et al., 2009
Siggeforasjön	Mesotrophic	0.76	11	Lindström, 1998
Ramsen	Mesotrophic	0.394	11.5	Hallgren et al., 1977

Sampling was carried out during winter and summer. Water was collected on March 9 and July 6 from Ekoln, March 14 and July 16 from Erken, March 14 and July 9 from Långsjön, March 16 and July 4 from Siggeforasjön, and March 20 and July 2 from Ramsen. For the winter sampling, all lakes were covered by ice and an ice drill was used to make holes for collection of water samples. Surface, middle, and bottom water samples were collected from sampling depths using a Ruttner water sampler (1 L and 2 L).

### Temperature, Oxygen, and Sampling Depths

On each sampling occasion, vertical temperature and oxygen concentration profiles were recorded at 1 m intervals using an Oxi 340i (WTW) probe.

### CH_4_ Concentrations

For CH_4_ samples, a rubber tube was connected to the Ruttner sampler, facilitating the transfer of water from the sampler into 125 ml infusion bottles without introducing air bubbles. Bottles were flushed with at least 2 volumes of water. Two NaOH pellets were then added to each bottle as a preservative. Red rubber stoppers with previously inserted needles (0.6 mm × 24 mm) were used to get rid of excess water from the head space of bottles and then needles were removed and bottles were subsequently sealed with 20 mm crimp seals. Samples were stored in an inverted upright position and kept at 4°C until analysis.

Samples were equilibrated to room temperature (20°C) prior to analysis by gas chromatography (GC) using an Agilent 7890A. The GC was calibrated with a dilution series of CH_4_ gas using a flame ionization detector. For sample analysis, a headspace was introduced by replacing 20 or 40 ml of the liquid with pure nitrogen gas while the bottle was placed upside-down in a three-finger clamp attached to a ring stand. Two replicates were analyzed for each water sample.

### Total Bacterial Abundance

Flow cytometry was used to estimate total bacterial abundance. Samples were preserved by adding borax-buffered and filter sterilized formaldehyde to 2% final concentration. For flow cytometry, 200 μl of each preserved sample was stained with 20 μl of 5 mM Syto 13 (Invitrogen^®^) for 10 min (del Giorgio et al., 1996). Cells were counted using a flow cytometer (Cyflow Space, Partec). Two replicates were analyzed for each sample.

### Water Characteristics

Dissolved organic carbon (DOC), pH, total phosphorous (TP), total dissolved phosphorus (TDP), dissolved organic matter absorbance, water color, and chlorophyll-*a*, were measured in surface, middle, and bottom water samples. Water was passed over 0.2 μm membrane filters (Pall Corp.) for analysis of DOC, TDP, PO_4_^3-^, SO_4_^2-^, water absorbance, and water color. Unfiltered water was used for analysis of TP. pH was measured immediately (1–3 h) after bringing samples to the laboratory using a pH meter (Philips PW 9420) with BlueLine electrode (SCHOTT Instruments). DOC was measured using a total organic carbon analyzer (TOC-5000, Shimadzu). Inorganic carbon was removed by acidification (final concentration of HCl was 0.01 M) and bubbling. Samples were oxidized to carbon dioxide (CO_2_) by high temperature catalytic combustion followed by IR-detection. Samples (6 ml each) were filled in acid washed-glass test tubes (provided) and 50 μl 1.2 M HCl was added to each sample and mixed by gentle vortexing. Known amount of potassium phosphate was used for calibration. TP and TDP were analyzed as previously described by Menzel and Corwin (1965). Phosphate (PO_4_^3-^) analysis was followed by Murphy and Riley (1962, 1968). Sulfate (SO_4_^2-^) ion was determined with ion chromatography (883 Basic IC plus-Metrohm). Absorbance scans (200 nm to 600 nm) were performed in a 5 cm quartz cuvette and measured in a Lambda 40 spectrophotometer (PerkinElmer). Water color was then derived from the absorbance reading at 436 nm as described in Broberg (2003). For chlorophyll-*a*, cells from 0.5 l of water were retained by filtration onto glass fiber filters (GF/F; Whatman). Filters were immediately frozen at -20°C until analysis and duplicates were separately extracted in 95% ethanol and measured in a spectrophotometer (Hitachi U-2000) as described by Jespersen and Christoffersen (1987).

### DNA Extraction

Between 300 and 500 ml of water was filtered through sterile 0.2 μm Supor membrane filters (Pall Corp.). Cells retained on filters were subsequently frozen at -80°C until DNA extraction using the PowerSoil^®^ DNA Isolation Kit (MoBio, Carlsbad, CA, USA). Prior to extraction, each membrane was cut into small pieces with a pair of scissors and then added to the PowerSoil^®^ Bead Tube. The extraction protocol was then followed as detailed in the instruction manual provided by the manufacturer. After DNA extraction, all samples were analyzed with 1% agarose gel electrophoresis to verify the quality of the extracted DNA. In addition, a picogreen assay was used to quantify extracted DNA (Quant-it^TM^ PicoGreen dsDNA Reagent Kit, Invitrogen) using an Ultra 384 fluorometer (Tecan) as recommended by the manufacturer.

### Polymerase Chain Reaction Amplification, Cloning, and Sequencing

The extracted DNA (mean 14 ng/μl) was used as template in PCR with *pmoA* primers A189F and mb661R (Costello and Lidstrom, 1999). This primer pair capture most groups of aerobic methane-oxidizing bacteria without amplification of equally abundant *amoA* genes (Bourne et al., 2001). PCR amplifications (for *pmoA*) were done in 20 μl reactions in 0.2 ml tubes using a thermal cycler (BioER). Each PCR mixture contained 2 mM MgCl_2_, 1× PCR buffer (Invitrogen), 0.2 mM dNTPs, 0.25 μM each of the forward and reverse primers (A189F/mb661R), 4% bovine serum albumin (New England Biolabs, USA), 0.05 units of Taq DNA Polymerase-Recombinant (Invitrogen), and 10-fold diluted DNA. All PCR reactions were performed along with one negative control containing DNase/RNase free water instead of DNA template. A thermocycling program with an initial denaturation step at 94°C for 3 min followed by 40 cycles of 94°C for 1 min, 55°C for 1 min and 72°C for 1 min, and a final extension at 72°C for 5 min was used. Primers used for PCR amplifications of *pmoA*, clone libraries and qPCR are listed in Supplementary Table S1. After amplification, 1% agarose gel electrophoresis was performed to verify correct amplification and size of the gene by comparison to a TrackIt 100 bp DNA Ladder (Invitrogen) (Supplementary Figure S2).

PCR products generated from DNA extracted from the bottom layer of two lakes (Ekoln and Ramsen), were cloned using the TOPO TA Cloning kit (Invitrogen) as previously described (Eiler and Bertilsson, 2004). Sanger sequencing was carried out at the Uppsala Genome Center using an ABI3730XL DNA Analyzer (Applied Biosystems). All nucleotide sequences were deposited at the National Center for Biotechnology Information (NCBI) under accession numbers KC588447–KC588466. A phylogenetic tree was constructed with Mega 5 using the maximum likelihood approach and Jukes-Cantor model. The topologies of the phylogenetic tree were calculated by bootstrap analysis with 1000 replications.

### qPCR Analysis

To determine the abundance of methanotrophs, qPCR was carried out using a Chromo 4 System (BIO-RAD). The 20 μl reactions contained 10 μl of 2× Master mix (KAPA SYBR^®^ FAST qPCR Master Mix Universal), 200 nM of each primer, 1 μl of DNA template, and DNase/RNase free water to a final volume of 20 μl. For each sample, reactions were carried out in duplicate whereas linearized plasmid standards were analyzed in triplicate. Three step cycling protocols were followed with an initial 3 min denaturation at 95°C followed by 40 cycles of denaturation at 95°C for 3 s, annealing at 60°C for 30 s, and extension at 72°C for 30 s. Fluorescence data was acquired at 72°C after completing each consecutive cycle. After 40 cycles, melting curve analysis was performed by raising the temperature from 55 to 95°C and reading the fluorescence 10 s after every 0.5°C increase in temperature. A 10-fold dilution series of known copies of linearized plasmid (pCR4-TOPO, Invitrogen) containing a single copy of *pmoA* gene (GenBank accession number KC588448) was used as a standard curve. The mean qPCR efficiency and dissociation/melting temperature (*T*_m_) were determined to 105% and 85°C, respectively. The coefficient of determination (*R*^2^) was 0.98. We assumed that each methanotrophs contained a single copy of the *pmoA* gene. The methanotrophs abundance was calculated as gene copy numbers per liter of water conservatively assuming 100% DNA extraction efficiency and the relative methanotrophs abundance was estimated by comparing with flow cytometry based estimates of total microbial abundance. The relative methanotroph abundance data were used for statistical analyses.

### T-RFLP Analysis

Terminal-restriction fragment length polymorphism (T-RFLP) analysis was performed on all extracted DNA samples. Fluorescently labeled gene fragments were generated by PCR using the mb661R primer with a hexachloro-6-carboxyfluorescein (A189-HEX) label. PCR conditions were the same as above. After PCR amplification, amplicons were purified using the QIAquick PCR purification kit (QIAGEN) and then loaded on the agarose gel to select for the expected amplicon fragment sizes and remove primer dimers (Supplementary Figure S2). DNA bands (*pmoA* amplicons) were excised from the gel and then gel-purified using the QIAquick gel extraction kit (QIAGEN). Quantification was subsequently carried out with the picogreen assay as described above. For each T-RFLP analysis, 40 ng of DNA was digested with 4 U of the restriction enzyme *Mse*I (New England Biolabs) for 16 h at 37°C according to the manufacturer’s instructions. After heat inactivation at 65°C for 20 min, all samples were stored at -20°C. Duplicates for each sample were sent to the Uppsala Genome Center for size separation by capillary electrophoresis using an ABI3730XL DNA Analyzer (Applied Biosystems) run in Genescan mode. The T-RFLP data were processed with GeneMarker V 2.20 (SoftGenetics). Terminal restriction fragments (T-RFs) between 50 and 500 bp in size were considered in the subsequent analysis. T-RFs differing ≤0.5 bp were considered to be the same fragment. Relative peak area was used as a measure of abundance and a 0.5% cut-off value was used as a baseline threshold.

### PLS Model and Statistics

Partial least squares regression (PLS) was used to relate and predict the relative methanotroph abundance to an extensive set of environmental parameters using XLSTAT. Data were square root transformed prior to the PLS analysis. In PLS, the model quality indices were reported as Q^2^(cum), R^2^Y(cum) and R^2^X(cum) parameters. The Q^2^(cum) index represents the global contribution of the components and identify the most stable model (maximum = 1). The R^2^Y(cum) and R^2^X(cum) represent the explanatory power of the components for the dependent variable and explanatory variables, respectively. The beta/standardized coefficient test was performed to understand which of the independent variables have greater effect on the dependent variable (methanotrophs). In addition, a goodness of fit statistics was performed to fit the model. In a PLS plot, variables are strongly correlated when they appear on the perimeter of the circle. For positive correlations, variables appear close to each other and for negative correlation, variables appear far from one another. Analysis of variance (ANOVA) was performed to test for significant differences in methanotroph abundance between winter and summer and between water depths (surface, middle, and bottom) as well as interactive effects. For T-RFLP data, a permutational multivariate analysis of variance (PERMANOVA) test (permutations = 1000) was performed in R using “vegan” package (Oksanen et al., 2013) along with Bray–Curtis distance matrix to identify significant differences in methanotrophic community composition by seasons, lakes, and depths.

## Results

### Environmental Conditions

**FIGURE 1:**
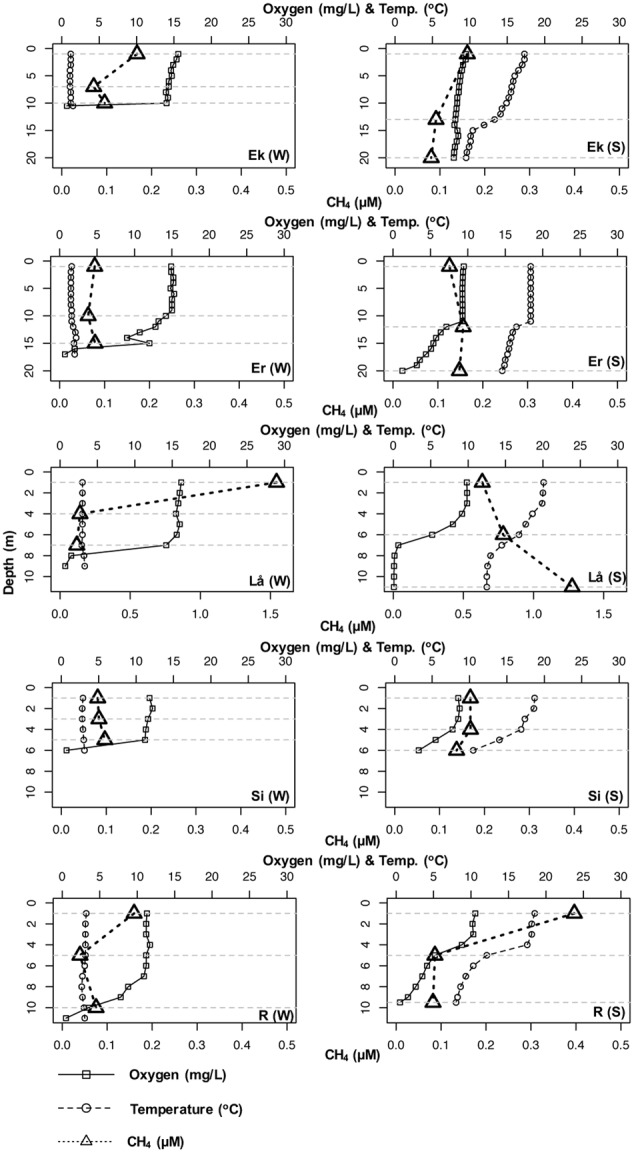
**Temperature (°C), dissolved oxygen (mg/l), and CH_4_ (μM) profiles of five Swedish lakes in winter (March 2012) and summer (July 2012).** Ek, Ekoln; Er, Erken; Lå, Långsjön; Si, Siggeforasjön; R, Ramsen; W, Winter; S, Summer. The dotted lines represent three sampling depths (surface, middle, and bottom).

All lakes were ice covered during the winter sampling and all had developed a distinct thermocline during the summer sampling. Except for Siggeforasjön which was moderately acidic (pH < 7), the lakes were slightly alkaline (pH > 7). For all lakes, dissolved oxygen concentration dropped down to <1.3 (mg/l) at the largest depths (**Figure 1**) except for summer samples of Ekoln (7.85 mg/l) and Siggeforasjön (3.22 mg/l). The bottom samples of Ekoln (Summer) were taken at 20 m as the sensor cables prevented us from sampling further down. CH_4_ concentration varied only slightly over depth and between seasons within each lake (**Figure 1**). The highest concentration of CH_4_ was detected in Långsjön during both seasons; surface samples in winter (1.53 μM) and bottom samples in summer (1.28 μM). **Figure 1** and **Table 2** summarize the physico-chemical properties of the five studied lakes in winter (March) and summer (July).

**Table 2:** Physico-chemical characteristics of the five study lakes for the two sampling occasions (winter and summer).

Lake	Depth	pH	DOC (mg/L)	Color (mg Pt/L)	Chl-a (μg/L)	TP (μg/L)	TDP (μg/L)	PO_4_^3-^ (μg/L)	CH_4_ (μM)	SO4^2-^ (mg/L)
Ekoln (Winter)	S	7.8	10.5	66	2	ND	41	28	0.17	30.11
	M	7.8	11.7	63	2	ND	35	27	0.07	31.91
	B	7.8	11.6	67	1	ND	33	24	0.09	33.69
Ekoln (Summer)	S	8.2	15.2	65	24	26	8	5	0.16	30.11
	M	7.9	14.5	63	6.5	26	13	5	0.09	29.74
	B	7.8	14	65	3.1	35	21	18	0.08	29.72
Erken (Winter)	S	8	6.4	14	3.5	ND	42	36	0.08	26.42
	M	8.1	9.4	16	2	ND	50	44	0.06	40.16
	B	7.7	8.7	17	ND	ND	51	41	0.08	35.32
Erken (Summer)	S	8.4	10.9	16	9.3	28	7	2	0.13	29.20
	M	8.1	10.9	17	4.8	22	18	10	0.16	28.40
	B	7.7	11.5	18	3.4	59	43	38	0.15	27.63
Långsjön (Winter)	S	8	3.7	8	7	ND	6	17	1.53	67.17
	M	8.1	3.8	6	8.4	ND	8	53	0.14	68.11
	B	7.8	3.4	6	7.6	ND	3	0	0.12	93.76
Långsjön (Summer)	S	8.6	7.4	8	4.8	16	0	3	0.64	71.02
	M	8.2	6.8	7	3.4	14	1	3	0.79	71.94
	B	7.8	5.9	7	2	19	5	6	1.28	68.25
Siggeforasjön (Winter)	S	6.4	18.9	137	0.6	ND	4	3	0.08	6.25
	M	6	17.4	140	1.4	ND	3	4	0.08	4.04
	B	6	19.9	139	0	ND	1	4	0.10	5.41
Siggeforasjön (Summer)	S	7.1	18.9	129	7.9	15	3	6	0.17	5.05
	M	6.9	19.7	134	1.1	9	5	3	0.17	5.23
	B	6.5	18.2	127	1.1	17	3	2	0.14	5.32
Ramsen (Winter)	S	7.7	13.1	46	9.8	ND	6	4	0.16	11.16
	M	7.8	11.2	48	3.4	ND	8	8	0.04	9.52
	B	7.4	14.9	54	0.3	ND	16	15	0.07	14.73
Ramsen (Summer)	S	7.9	16.4	49	8.4	25	8	2	0.40	10.85
	M	7.4	15.3	50	3.4	13	7	0	0.09	10.96
	B	7.3	16.1	53	1.4	23	13	3	0.08	11.43

*S, Surface; M, Middle; B, Bottom; DOC, dissolved organic carbon; Chl-a, chlorophyll-a; TDP, total dissolved phosphate; TP, total phosphate; ND, Not determined.*

### Total Bacterial Abundance and Methanotrophs in Lakes

**FIGURE 2:**
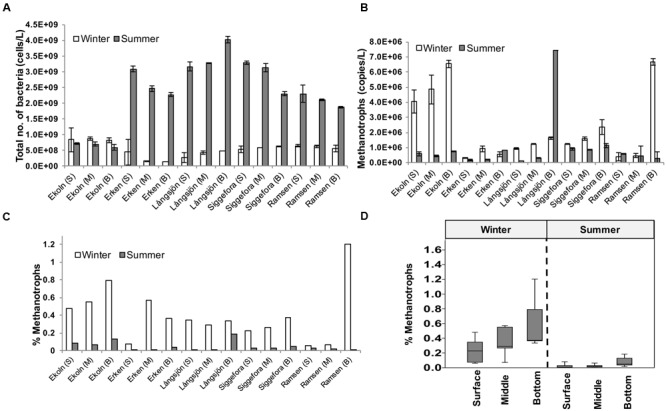
**Bacterial and methanotroph abundance.** Bacterial **(A)** and methanotroph abundance **(B)** in five Swedish lakes in winter and summer. Ratio (%) of methanotrophs is compared to total bacterial abundance **(C)**. **(D)** Box plot showing percent of methanotrophs in surface (S), middle (M), and bottom (B) waters from the five studied lakes in winter and summer.

Except for lake Ekoln, bacterial abundance was typically more than twofold higher in summer samples than in winter (**Figure 2A**). The highest number of bacteria was observed in bottom samples from the summer sample of lake Långsjön at 4.0 (±0.1) × 10^9^ cells/l while the lowest bacterial abundance was observed in Erken winter samples at 2.5 (±1.7) × 10^8^ cells/l.

Methanotroph abundance detected by qPCR as *pmoA* gene copies ranged from 10^5^ to 10^6^/l (**Figure 2B**). The highest abundance of methanotrophs was observed in summer samples of deep water from lake Långsjön at 7.4 × 10^6^ ± 1.6 × 10^4^ (mean ± SD) *pmoA* gene copies/l and for deep lake Ramsen winter samples at 6.7 × 10^6^ ± 2.2 × 10^5^. The lowest number of methanotrophs was observed in winter samples from lake Erken at 6 × 10^5^ ± 8.4 × 10^4^ gene copies/l. All winter samples from Ekoln showed high methanotroph concentrations. The ratio between methanotrophs and total number of bacteria was less than 1.3% for both seasons and was always higher in winter when compared to the corresponding summer samples (**Figure 2C**). On average, the bottom samples also featured higher methanotroph contribution (0.35%), compared to the middle (0.2%) and surface (0.13%) samples (**Figure 2D**), but the ANOVA only identified the seasonality effect as significant (ANOVA *F* = 24.81, *p* < 0.001) while the effect of water layer was weaker (*F* = 3.27, *p* = 0.055).

### Relationships between Environment Parameters and Methanotrophs

**FIGURE 3:**
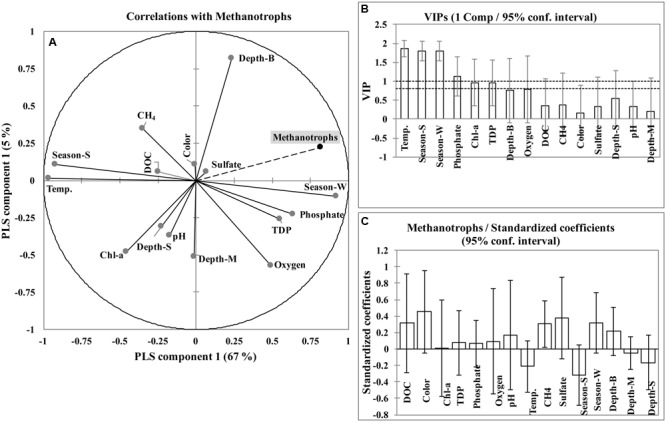
**Correlation circle of partial least squares (PLS) regression, illustrating the correlations of the variables with the first two axes (associated to the first two components) (A).** The VIPs (Variable Importance for the Projection) for each explanatory variables of first component **(B)**. Standardized/beta coefficient test from PLS model and variables are not significant when confidence interval around the standardized coefficient include 0 **(C)**. Season-W, winter season; Season-S, summer season; DOC, dissolved organic carbon; TDP, total dissolved phosphorous; Chl-a, chlorophyll-*a*; Depth-S, depth (surface); Depth-M, depth (middle); Depth-B, depth (bottom).

In the PLS model, the relative abundance of methanotrophs was mainly explained by component 1 (67%) and to a lesser extent by component 2 (5%). The first component (Comp1) was calculated with the Q^2^(cum), R^2^Y(cum), and R^2^X(cum) parameters of 0.59, 0.67, and 0.27, respectively and the second component (Comp2) was calculated with the Q^2^(cum), R^2^Y(cum), and R^2^X(cum) parameters of 0.49, 0.72, and 0.41, respectively (Supplementary Figure S3). The quality of components assessed through cross validation showed that only component 1 (Q^2^ = 0.59) was significant (Q^2^ limit > 0.097, corresponding to *p* < 0.05). The relative methanotroph abundance was positively correlated with winter season (*r* = 0.76), bottom layer (*r* = 0.32), phosphate (*r* = 0.47), TDP (*r* = 0.4), dissolved oxygen (*r* = 0.33), SO_4_^2-^ (*r* = 0.14) and color (*r* = 0.07); and negatively correlated with summer season (*r* = -0.76), temperature (*r* = -0.78), chlorophyll-*a* (*r* = -0.41), surface layer (*r* = -0.23), CH_4_ (*r* = -0.16), DOC (*r* = -0.15), pH (*r* = -0.14), and middle layer (*r* = -0.1) (**Figure 3A**). The VIPs (Variable Importance for the Projection) for each explanatory variable of both components (Comp1 and Comp2) showed that temperature, season, and phosphate contributed the most to the model (VIP > 1; **Figures 3B** and Supplementary Figure S4). In the PLS model, the standardized coefficient test was done to identify important predictor(s). Only CH_4_ was significant (*p* < 0.05) as the coefficient was different from 0 (**Figure 3C**). Goodness of fit statistics showed that methanotroph abundance was accurately predicted by the model (R^2^ = 0.808, SD = ±0.126).

### *pmoA* Clone Libraries and Phylogenetic Analysis

**FIGURE 4:**
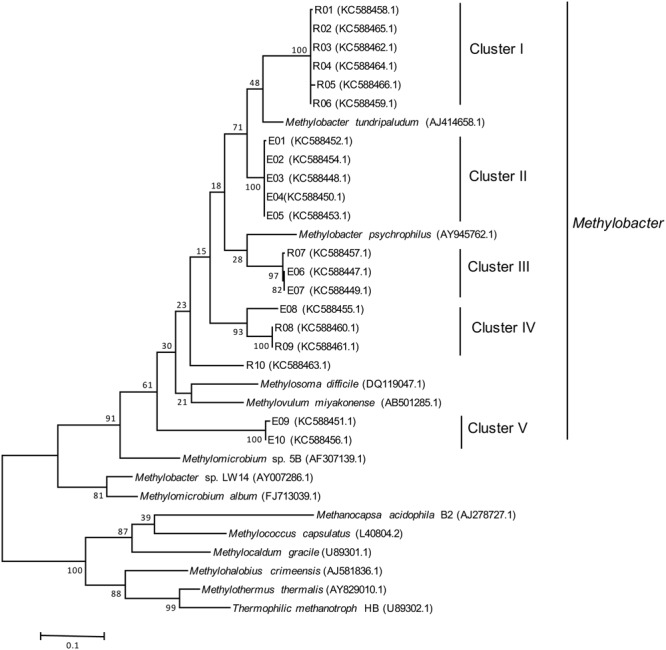
**Maximum likelihood phylogenetic tree of *pmoA* gene sequences (508 bp) amplified by A189F/mb661R from two clone libraries (10 clones from Ekoln and 10 clones from Ramsen).** Number of bootstrap replications is 1000.

Two separate clone libraries were generated from the *pmoA* gene fragments amplified from DNA of the bottom water samples of Ekoln and Ramsen. From each lake, the inserts from 10 randomly selected clones were sequenced, confirming the presence of methanotrophs in the lakes (**Figure 4**). All sequences obtained were affiliated with *Methylobacter*, and overall grouped into five phylogenetic clusters (clusters I–V, **Figure 4**). Six clones from Ramsen (R01–R06) grouped in cluster I while two clusters were unique for Ekoln; cluster II (E01–E05) and cluster V (E09, E10). Cluster III (R07, E06, E07) and cluster IV (E08, R08, R09) were mixed clones from both Ekoln and Ramsen.

### Methanotroph Community Characterization

**FIGURE 5:**
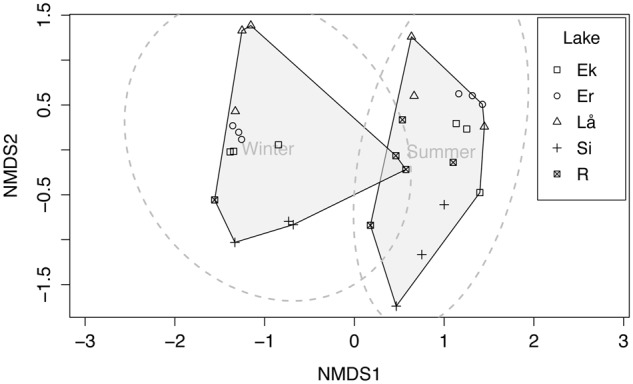
**Two dimensional non-metric multidimensional scaling (NMDS) plot of methanotrophic bacterial terminal restriction fragments (T-RFs) at three depths and two seasons (winter and summer) of five Swedish lakes in winter and summer.** The stress value = 0.153. Ek, Ekoln; Er, Erken; Lå, Långsjön; Si, Siggeforasjön; R, Ramsen. Circles (gray dotted line) represent 95% confidence interval and shaded regions represent winter and summer samples.

T-RFLP data were analyzed to detect differences in methanotroph community composition between lakes, seasons, and depth layers. The non-metric multidimensional scaling (NMDS) plot based on Bray–Curtis indices clearly identified two community types (winter and summer) at 95% confidence interval (**Figure 5**). In general, summer samples seemed to be more diverse than winter samples (i.e., more T-RFs), and especially summer samples (surface) of Erken and Långsjön showed >75 unique T-RFs (Supplementary Figure S5). Adonis test (PERMANOVA) showed that lakes (*F* = 2.922, *p* < 0.01), seasons (*F* = 8.45, *p* < 0.001), and interaction between lakes and seasons (*F* = 2.66, *p* < 0.01) had a significant effect on community composition.

## Discussion

### Methanotrophs and CH_4_ in Lakes

Methanotrophs were present in all the studied lakes regardless of depth (surface, middle, and bottom), season (winter and summer), trophic status (Eutrophic: Ekoln; Mesotrophic: Erken, Siggeforasjön, Ramsen; and Oligotrophic: Långsjön) as well as lake water chemistry. This suggests that methanotrophs are ubiquitous and an integral part of the lake ecosystem. CH_4_ is by definition a critical resource for methanotrophs. The average CH_4_ concentration of our studied lakes were 0.19 and 0.29 μM for summer and winter, respectively. Similar concentrations were previously observed in 13 Swedish lakes and seven ice-covered Swedish lakes where surface CH_4_ concentrations ranged from 0.08 to 1.9 μM (Bastviken et al., 2004) and 0.04 to 1.11 μM, respectively (Denfeld et al., 2016). Although, the bottom water CH_4_ concentrations were much lower in our lakes compared to previously reported Finish lakes, where the average CH_4_ concentration was 20.6 μM (Juutinen et al., 2009).

Among the lakes, the bottom layer of Långsjön was identified as a potential “hot spot” for both CH_4_ and methanotrophs. This lake surprisingly had among the lowest DOC (5.2 mg/l) and highest SO_4_^2-^ concentrations (67–94 mg/l) compared to the others and our results are thus in agreement with a previous study suggesting that surface CH_4_ and DOC are inversely correlated (Bastviken et al., 2004). This particular lake is groundwater fed and drain a catchment dominated by soils of marine origin (Denfeld et al., 2016) and also featured lower oxygen levels at the bottom compared to the other studied lakes, likely creating conditions conducive for CH_4_ production. However, a recent microcosm study (Denfeld et al., 2016) performed in the laboratory under variable CH_4_ concentrations (1, 10, 100, 500 μM) and constructing temperatures (2, 4, 10, 20°C) could not demonstrate any significant CH_4_ oxidation in surface waters from this lake. The probable causes of this observation could be interference from other controlling factors such as the presence of alternative electron acceptors (e.g., SO_4_^2-^_,_ NO_3_^-^_,_ etc.) or spatial patchiness of resident methanotroph communities.

### Environmental Control of Methanotrophs

The relative methanotroph abundance was significantly higher in winter than in summer for all lakes (**Figure 2C**) while total bacterial abundance was higher in summer than in winter (**Figure 2A**). This implies that the lower temperatures in winter combined with other prevailing conditions such as minimal inputs of phytoplankton-derived organic substrates is a conducive environment for methanotrophs while other bacteria are less competitive. This agrees with previous studies reporting that the largest methanotroph biomass and activity in lakes occurred at low temperature (Sundh et al., 2005). In our study, methanotrophs were detected throughout the water columns and at higher abundances at the bottom of the lakes, suggesting that methanotrophs are widely distributed in temperate lakes and that they preferentially grow in sub-oxic conditions.

CH_4_ is known to be an important regulator that affects methanotroph abundance. CH_4_ was found to be a significant variable, but showed a weak and inverse correlation (*r* = -0.16) with relative methanotroph abundance. This suggests that methanotrophs utilized CH_4_ as their energy source and hence methanotrophs proliferated while depleting CH_4_ from the water column.

Oxygen is also a critically important factor controlling the growth and abundance of aerobic methanotrophs. For surface waters, we observed higher dissolved oxygen in winter and this was associated with a higher representation of methanotrophs in the community. This is in agreement with earlier studies showing that plant root systems provide high amounts of oxygen to the rhizosphere (van der Nat and Middelburg, 1998; Popp et al., 2000) enhancing the growth, abundance, and activity of methanotrophs (Yun et al., 2012). Additionally, the availability of free PO_4_^3-^ appeared to be strongly coupled to methanotroph abundance. Similar observations were reported by Denfeld et al. (2016) who suggested that a shortage of labile phosphorus may hamper methanotrophs by invoking stronger competition with other bacterial groups with higher affinity for this critical nutrient.

### Identity and Community Structure of Methanotrophs

The sequence analysis of 20 randomly selected *pmoA* clones from bottom winter samples of two lakes (Ekoln and Ramsen) revealed a significant representation of five clusters of *Methylobacter* affiliated with Type I methanotrophs of class gammaproteobacteria. This represents one out of 14 genera of methanotrophs (Jiang et al., 2010). Similar results have also been observed in other studied temperate lakes where Type I methanotrophs dominated (Sundh et al., 2005; Denfeld et al., 2016; Ricão Canelhas et al., 2016).

The diversity of methanotrophs and their spatial and temporal distribution patterns and dynamics are not well understood in temperate lakes. Our T-RFLP results revealed two distinct clusters of methanotrophic communities in temperate lakes, suggesting seasonal turnover of this microbial guild. We observed comparatively higher numbers of T-RFs in summer samples, suggesting a more evenly composed methanotroph community under such conditions. Methanotrophs were nevertheless highly abundant in winter, but the extended period of low-temperature and assumed limited dynamic shifts in the planktonic food webs during winter seem to have favored a few populations able to cope with and profit from such conditions. Low predation in winter may have played a role in this regard and could potentially open up for expansion of particularly competitive methanotroph populations, such as some previously identified Type I methanotrophs (Henckel et al., 2001). Our results also indicate significant differences of methanotrophic communities across lakes of different trophic status while there were no significant differences in methanotroph community composition between the different depth layers in the respective lake, implying either significant vertical scrambling of the communities or wide local niches with regards to oxygen, temperature, and water chemistry.

In conclusion, we uncovered a dynamic distribution of methanotroph communities between lakes, both with regards to abundance and predominant populations and communities. CH_4_ concentrations were overall low in the studied lake ecosystems and was inversely correlated with methanotroph abundance likely due to CH_4_ being depleted by the methanotrophs.

## Author Contributions

MSS and SB designed the project. MSS carried out the field work and laboratory analyses with assistance and advice from SB. MSS analyzed the data and drafted the manuscript. A final version of the MSS was then prepared by MSS and SB.

## Conflict of Interest Statement

The authors declare that the research was conducted in the absence of any commercial or financial relationships that could be construed as a potential conflict of interest.
