# Supplementary material for: Seasonal Variation in Abundance and Diversity of Bacterial Methanotrophs in Five Temperate Lakes
Source: Front Microbiol. 2017 Feb 3;8:142. doi: 10.3389/fmicb.2017.00142 (PMC5289968; doi:10.3389/fmicb.2017.00142)
Supplement: Supplementary file 1 [file Data_Sheet_1.pdf]

Supplementary Material

Seasonal variation in abundance and diversity of bacterial methanotrophs in five temperate lakes

Md Sainur Samad, Stefan Bertilsson*

*** Correspondence:** Stefan Bertilsson, stebe@ebc.uu.se

1 Supplementary Figures and Tables

1.1 Supplementary Figures

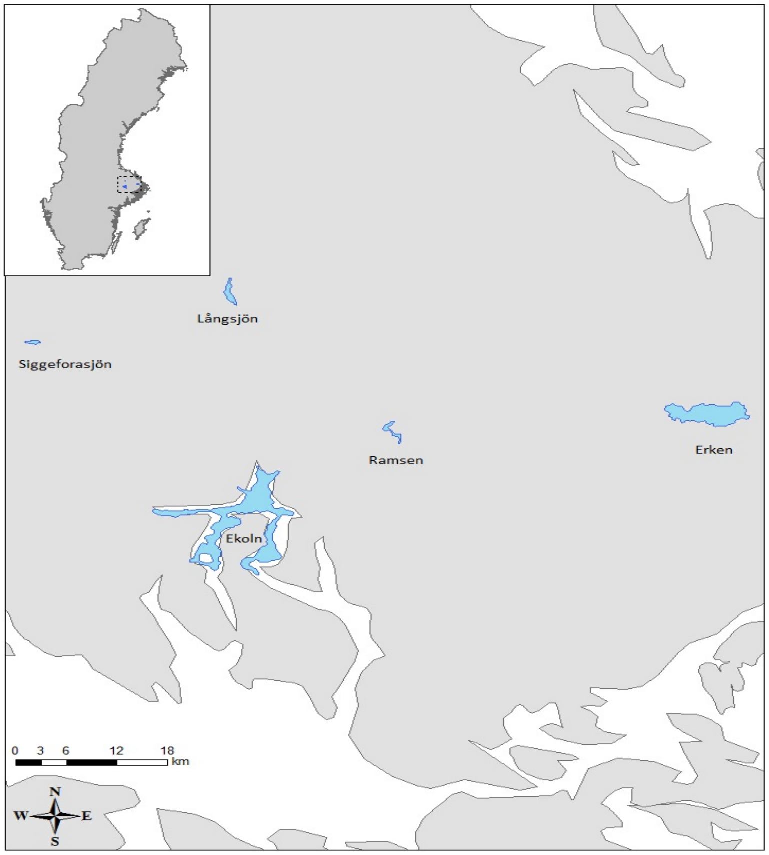

Supplementary Figure S1 Location of the five Swedish lakes: Ekoln (59°46'N, 17°37'E), Erken (59°50'N, 18°35'E), Långsjön (60°01'N, 17°34'E), Siggeforasjön (59°58'N, 17°09'E) and Ramsen (59°49'N, 17°54'E) in Uppland, Sweden.

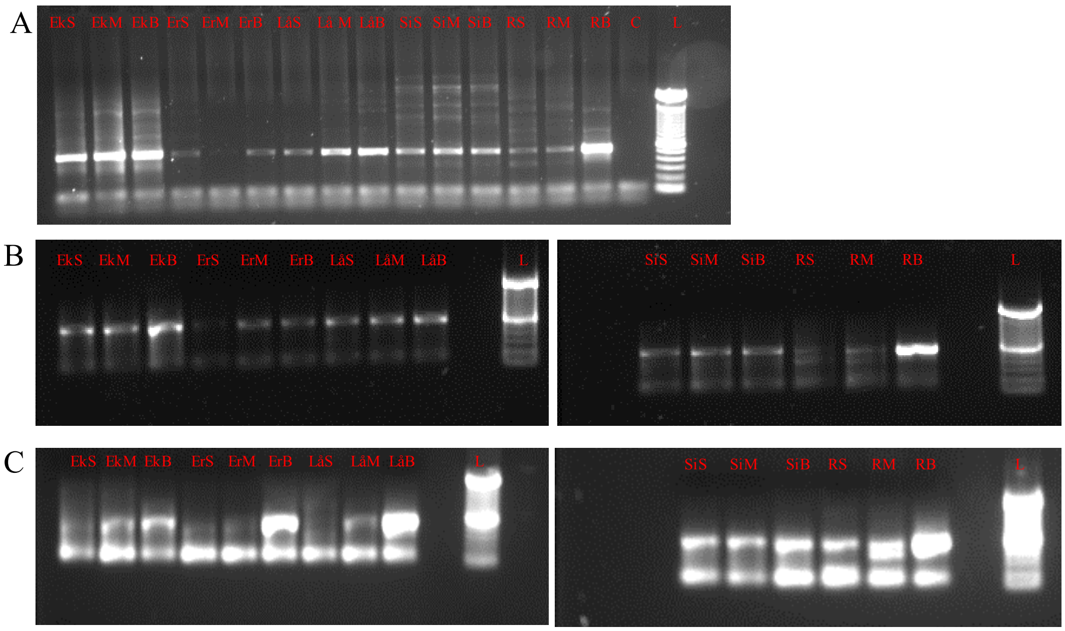

Supplementary Figure S2 Agarose gel of PCR products (*pmoA* gene amplification without florescence primers [A189F, mb661R]) from all winter (A) samples. (B & C) PCR products (*pmoA* gene amplification with florescence primers [A189-HEX, mb661R]) from winter (B) and summer (C) samples. PCR amplicons were loaded on agarose after PCR purification kit (B & C). Abbreviations of Ek, Er, Lå, Si, R, S, M, B, C and L represent Ekoln, Erken, Långsjön, Siggeforasjön, Ramsen, surface, middle, bottom, negative control, and DNA ladder (TrackIt 100bp DNA Ladder) respectively.

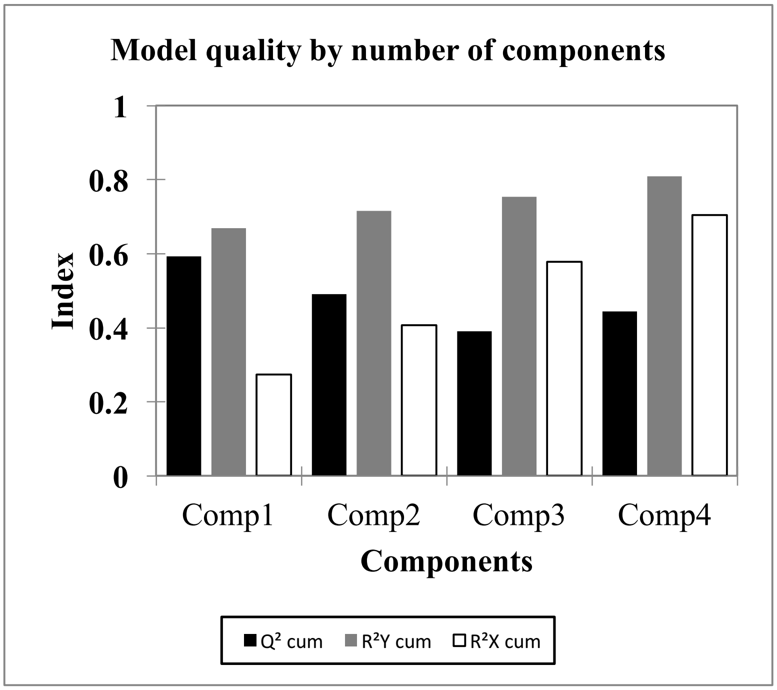

Supplementary Figure S3 PLS model quality by number of components (Comp 1 to 4).

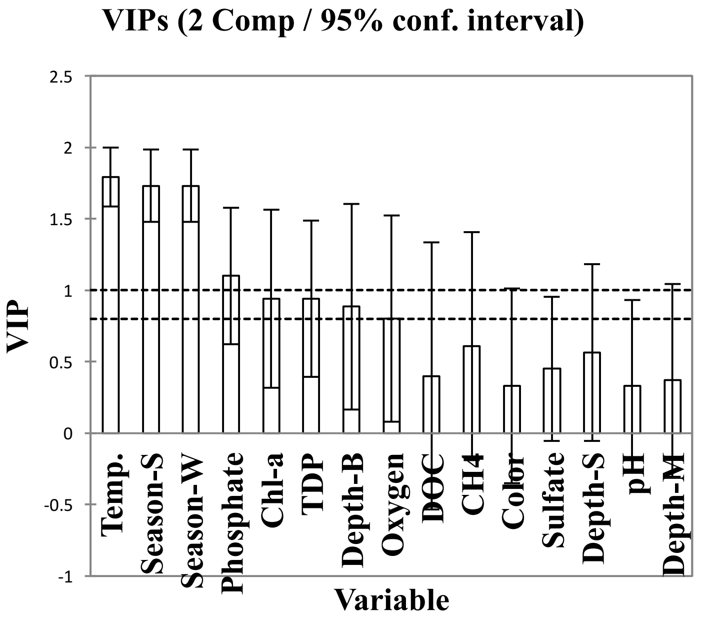

Supplementary Figure S4 The VIPs (Variable Importance for the Projection) for each explanatory variables of second component.

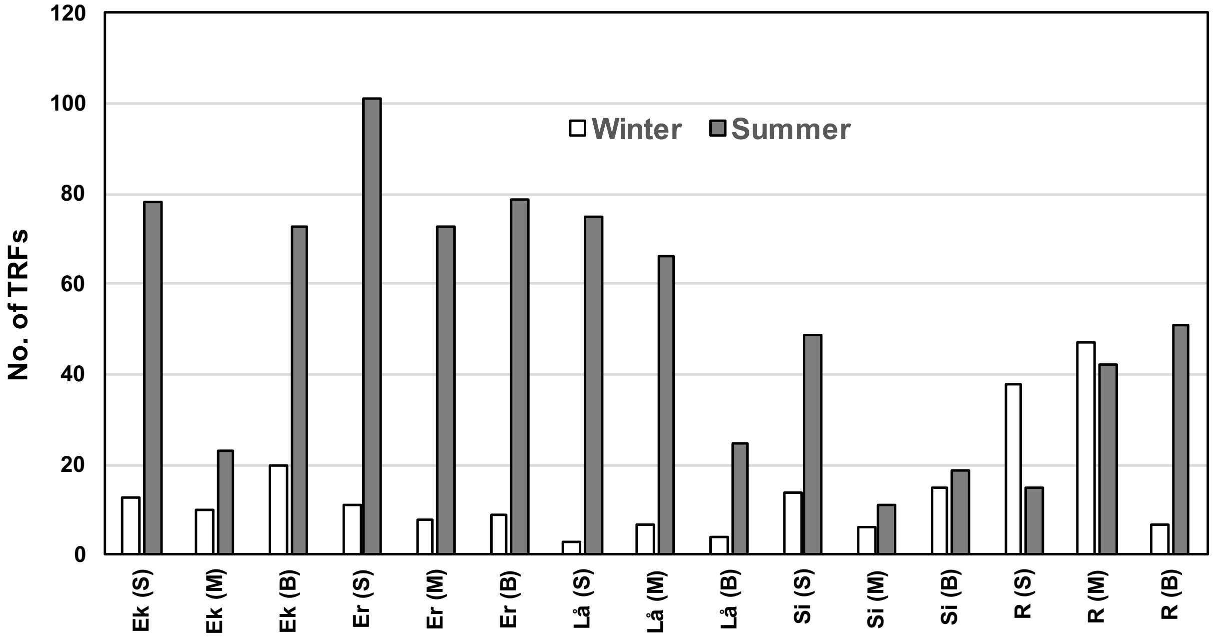

Supplementary Figure S5 Total number of Terminal Restriction fragments (TRFs) of Methanotrophs in five Swedish lakes. Ek = Ekoln , Er = Erken, Lå = Långsjön , Si = Siggeforasjön, R = Ramsen, S = Surface, M = Middle and B = Bottom.

1.2 Supplementary Table

Supplementary Table S1 Primers used in this study

	Forward/ reverse primer	Target group	PCR length (bP)	Sequence (5’ to 3’)[a]	Reference
pmoA	A189F/ mb661R	All methanotrophs	508	GGNGACTGGGACTTCTGG/ CCGGMGCAACGTCYTTACC	Costello and Lidstrom, 1999
**Sequencing primers**	M13F (-20)/ M13R	Inserted gene (*pmoA*) of *E. coli*		GTAAAACGACGGCCAG/ CAGGAAACAGCTATGAC	

[a] N, bases A, C, T, or G; M, bases A or C; S, bases G or C; Y, bases C or T

2 Reference

Costello, A. M., and Lidstrom, M. E. (1999). Molecular characterization of functional and phylogenetic genes from natural populations of methanotrophs in lake sediments. *Appl. Environ. Microbiol.* 65, 5066–5074.
